# Digitale Gesundheitsanwendungen (DiGA): Bewertung der Erstattungsfähigkeit mittels DiGA-Fast-Track-Verfahrens im Bundesinstitut für Arzneimittel und Medizinprodukte (BfArM)

**DOI:** 10.1007/s00103-021-03409-7

**Published:** 2021-09-16

**Authors:** Wolfgang Lauer, Wiebke Löbker, Barbara Höfgen

**Affiliations:** Bundesinstitut für Arzneimittel und Medizinprodukte (BfArM), Kurt-Georg-Kiesinger-Allee 3–5, 53175 Bonn, Deutschland

**Keywords:** App auf Rezept, BfArM, Digitale Gesundheitsanwendungen, DiGA, DVG, App on prescription, BfArM, Digital health apps, DiGA, DVG

## Abstract

Mit dem Digitale-Versorgung-Gesetz (DVG) hat der Gesetzgeber digitalen Gesundheitsanwendungen, kurz DiGA, einen Einzug in die Regelversorgung nach dem Fünften Buch Sozialgesetzbuch (SGB V) ermöglicht. Voraussetzung für die „App auf Rezept“ ist die Listung im DiGA-Verzeichnis nach positiv durchlaufenem Bewertungsverfahren beim Bundesinstitut für Arzneimittel und Medizinprodukte (BfArM). Dafür ist von den Herstellern neben umfangreichen Qualitäts- und Sicherheitsparametern auch ein positiver Versorgungseffekt nachzuweisen.

Mit dem DiGA-Verzeichnis bietet das BfArM seit Oktober 2020 adressatengerechte umfassende Transparenz zu den DiGA und deren Eigenschaften. Der Artikel erläutert den Weg zur „App auf Rezept“ von den Unterstützungs- und Beratungsangeboten durch das BfArM über den Ablauf des Bewertungsverfahrens (DiGA-Fast-Track) und die Bewertungskriterien bis hin zu den Inhalten des Verzeichnisses. Es zeigt sich, dass das Interesse an dem Fast-Track-Verfahren groß ist. Der Nachweis der positiven Versorgungseffekte, also eines tatsächlichen Mehrwerts für die Patienten, ist ausbalanciert und mit überwiegend randomisierten kontrollierten Studiennachweisen auf einem angemessenen Niveau. Dass das Verfahren auch mit Herausforderungen für die Antragsteller verbunden sein kann, z. B. Mängel adäquat in dem gesetzlich vorgegebenen Bewertungszeitraum zu adressieren, zeigen die Zahlen zurückgenommener Anträge im Verhältnis zu den gelisteten DiGA an. Das BfArM steht zu diesem neuen Verfahren in engem Austausch mit allen Beteiligten. Welche Überlegungen und Potenziale sich daraus für die Weiterentwicklung aus Sicht des BfArM ergeben, zeigt der Ausblick.

## Abstract (en)

With the Digital Health Care Act (DVG), the legislator has made it possible for digital health applications (DiGA) to be included in standard care and reimbursement in accordance with the Fifth Book of the German Social Code (SGB V). The prerequisite for the “app on prescription” is a listing in the DiGA directory after a positive evaluation procedure at the Federal Institute for Drugs and Medical Devices (BfArM). In addition to comprehensive quality and safety parameters, the manufacturers must also prove a positive healthcare effect.

With the DiGA directory, the BfArM has been offering addressee-oriented comprehensive transparency on DiGA and their properties since October 2020. The article explains the path to the app on prescription from the support and advisory services offered by the BfArM to the evaluation procedure (DiGA Fast Track) and the evaluation criteria to the contents of the directory. It can be seen that there is great interest in the Fast Track procedure. The evidence of positive healthcare effects, i.e. an actual added value for patients, is balanced and at an appropriate level with predominantly randomised controlled trial evidence. The fact that the procedure can also be associated with challenges for the applicants, e.g. to adequately address deficiencies in the legally prescribed assessment period, is indicated by the numbers of withdrawn applications in relation to the listed DiGA. The BfArM is in close exchange with all parties involved regarding this new procedure. The conclusion of this article shows which considerations and potentials result from this for further development from the point of view of the BfArM.

## Einleitung

Digitale Anwendungen (z. B. Apps) finden immer mehr Verbreitung im Alltag und auch in der Gesundheitsversorgung gewinnen sie zunehmend an Bedeutung. Neben den damit verbundenen technischen Möglichkeiten wächst auch das Angebot digitaler Unterstützung. Da eine systematische, unabhängige und transparente wissenschaftliche Bewertung zu den auf dem Markt verfügbaren Anwendungen bisher nicht vorlag, wuchs mit der Produktvielfalt auch der Bedarf an entsprechender Bewertung und vergleichender Übersicht als Orientierung für eine sinnvolle Therapiebegleitung [1, 2].

Der digitalen Transformation, aber eben auch diesen Anwenderwünschen Rechnung tragend hat der Gesetzgeber unter anderem mit dem Digitale-Versorgung-Gesetz (DVG) und der ergänzenden Rechtsverordnung, der Digitalen-Gesundheitsanwendungen-Verordnung (DiGAV), den Weg zur Erstattung digitaler Gesundheitsanwendungen (DiGA) durch die Gesetzliche Krankenversicherung (GKV) bereitet. Mit der „App auf Rezept“ wurde damit insgesamt einen Digitalisierungsschub im deutschen Gesundheitssystem bewirkt.

Um die Erstattungsfähigkeit von DiGA zu prüfen, wurde beim Bundesinstitut für Arzneimittel und Medizinprodukte (BfArM) ein speziell auf diese Anwendungen zugeschnittener Bewertungsprozess eingeführt: das sogenannte DiGA-Fast-Track-Verfahren. Nach erfolgreich durchlaufenem Verfahren wird die DiGA in das DiGA-Verzeichnis des BfArM aufgenommen.

Der Werdegang des Fast-Track-Verfahrens, der Bewertungsprozess selbst mit Kriterien bzw. Voraussetzungen und die Inhalte des DiGA-Verzeichnisses werden im Folgenden dargestellt. Die Beratungs- und Unterstützungsangebote des BfArM für DiGA-Hersteller werden vorgestellt und abschließend Erfahrungen und Ergebnisse aus den bisherigen Beratungs- und Bewertungsverfahren berichtet. Zudem wird ein Ausblick auf die Weiterentwicklung des Verfahrens gegeben.

## Der Weg zum DiGA-Fast-Track

Das BfArM ist u. a. für die Risikobewertung bei Medizinprodukten zuständig und beschäftigt sich bereits seit vielen Jahren intensiv mit verschiedenen Themen rund um die Digitalisierung bei Medizinprodukten und deren sichere und zuverlässige Anwendung.

So hat das BfArM beispielsweise bereits 2014 auf seiner Website eine Orientierungshilfe zur Frage veröffentlicht, wann Apps und andere Softwareanwendungen als Medizinprodukte anzusehen sind [3]. Dialogveranstaltungen adressierten den gesamten Weg der Medical App von der Idee über die Einordnung als Medizinprodukt und den entsprechenden Weg in den Markt bis hin zu Fragen der Kostenerstattung [4, 5].

Auf Basis der Erfahrungen aus vielen Anfragen und Gesprächen mit Entwicklern und Start-ups gründete das BfArM im Jahr 2017 sein Innovationsbüro als ergänzendes Beratungsangebot neben dem Scientific Advice, dem vor allem wissenschaftlichen Beratungsverfahren des BfArM zu Arzneimitteln und Medizinprodukten [6].

Darüber hinaus hat das BfArM weitere Themen rund um die Digitalisierung von Medizinprodukten aufgegriffen und z. B. in einer Dialogveranstaltung gemeinsam mit Hackern, Herstellern, Klinikbetreibern und dem Bundesamt für Sicherheit in der Informationstechnik (BSI) aktuelle Herausforderungen rund um das Thema Cybersicherheit von Medizinprodukten diskutiert [7].

Vor diesem Hintergrund hat sich das BfArM intensiv in die Initiative des Bundesministeriums für Gesundheit (BMG) zur Gestaltung und Etablierung des neuen DiGA-Fast-Track eingebracht. Gemeinsam wurden die Regelungen im DVG [8] wie auch weitere Details zur konkreten Ausgestaltung in der DiGAV [9] erarbeitet und z. B. im Rahmen der „DVG Startup Roadshow“ (Veranstaltungsreihe an verschiedenen Standorten in Deutschland) gemeinsam mit dem „health innovation hub“ (hih) des BMG und vieler weiterer Veranstaltungen die geplanten Regelungen frühzeitig erläutert und diskutiert.

## Der DiGA-Fast-Track

Mit der Einführung eines neuen, auf maximal 3 Monate begrenzten Bewertungsverfahrens beim BfArM hat der Gesetzgeber einen tatsächlichen Fast-Track zur Einbindung von DiGA in die Gesundheitsversorgung in Deutschland geschaffen, auf dessen Basis DiGA in kurzer Zeit neutral, verlässlich und wissenschaftlich fundiert bewertet werden. Bereits am 06.10.2020, und damit weniger als 6 Monate nach Inkrafttreten der DiGAV, wurden die ersten DiGA in das öffentlich zugängliche DiGA-Verzeichnis aufgenommen.

### Was ist eine DiGA? Zentrale Voraussetzungen

Bei digitalen Anwendungen, die z. B. als Apps Vitaldaten kontinuierlich erfassen und auswerten, sind im Wesentlichen Produkte mit medizinischer Zweckbestimmung von solchen für Lifestyleaspekte zu unterscheiden [3].

**Figure 1:**
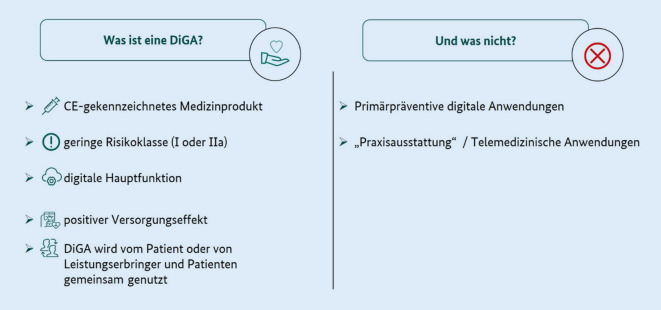

Der Gesetzgeber hat durch die Definition einer DiGA in § 33a SGB V Absatz 1 und 2 nur Produkte mit medizinischer Zweckbestimmung, also Medizinprodukte adressiert. Eine DiGA ist also grundsätzlich eine bereits rechtmäßig in Verkehr gebrachte Software oder ein anderes auf digitalen Technologien basierendes Produkt mit medizinischer Zweckbestimmung, also ein CE-gekennzeichnetes Medizinprodukt mit geringem Risikopotenzial (Risikoklasse I oder IIa nach der europäischen Medical Device Regulation (MDR) [10] bzw. im Rahmen der entsprechenden Übergangsvorschriften). Eine DiGA darf dabei nicht lediglich dem Auslesen oder Steuern eines Gerätes dienen, sondern muss „aktiv“ die Erkennung, Überwachung, Behandlung oder Linderung von Krankheiten, Verletzungen oder Behinderungen unterstützen. Primärpräventive Ansätze sind aufgrund anderer sozialrechtlicher Abrechnungswege ausgeschlossen (Abb. 1).

Im Rahmen der Anwendung einer DiGA können grundsätzlich auch Dienstleistungen wie Beratungen oder andere ärztliche oder psychotherapeutische Leistungen angeboten werden. Die Hauptfunktion muss allerdings klar durch die digitale Komponente zustande kommen und somit auch der nachzuweisende positive Versorgungseffekt auf der Anwendung der DiGA selbst und nicht auf begleitenden menschlichen Leistungen beruhen. Prinzipiell kann eine DiGA auch mit anderer Hardware (u. a. Sensoren, Wearables) interagieren bzw. diese umfassen. Auch hier gilt, dass dies nur zulässig ist, solange die Hauptfunktion überwiegend digital ist. Eine DiGA muss sich direkt an Patienten richten und überwiegend von diesen allein bzw. zusammen mit dem behandelnden Arzt oder Psychotherapeuten genutzt werden. Rein telemedizinische Ansätze oder digitale Anwendungen, die nur von Ärzten und Pflegenden z. B. zur Diagnostik oder Verlaufskontrolle genutzt werden („Praxisausstattung“), stellen keine DiGA im Sinne des § 33a SGB V dar.

### Das DiGA-Fast-Track-Verfahren im BfArM

**Figure 2:**
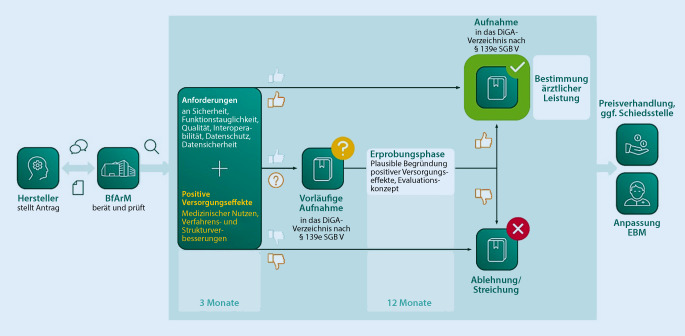

Damit DiGA den Weg in die Regelversorgung der gesetzlichen Krankenversicherung (GKV) finden, werden sie auf Antrag eines Herstellers beim BfArM dem Bewertungsverfahren unterzogen (Abb. 2). Die Anforderungen und die für die Bewertung des BfArM zu erbringenden Nachweise sind in § 139e SGB V sowie weiter konkretisierend in der DiGAV dargelegt. Die Bewertungszeit beträgt maximal 3 Monate nach vollständigem Antragseingang, ohne Möglichkeit eines Clock Stop (Anhalten der Bewertungsfrist z. B. während Nachlieferungen des Antragstellers).

Liegen noch keine umfassenden Daten zu „positiven Versorgungseffekten“ (siehe unten) vor, kann der Hersteller zunächst eine vorläufige Aufnahme in das DiGA-Verzeichnis beantragen. Hierzu bedarf es einer aussagekräftigen, plausiblen Begründung anhand systematischer Datenauswertungen mit der DiGA als Teil eines Evaluationskonzepts dafür, dass die DiGA einen Beitrag zur Verbesserung der Versorgung leistet und die entsprechenden Nachweise im Rahmen einer Erprobungsphase generiert werden können. Werden diese Nachweise erbracht, erfolgt die dauerhafte Aufnahme; gelingt dies nicht, wird die DiGA – mit Veröffentlichung der Begründung – aus dem Verzeichnis gestrichen.

Parallel prüft das BfArM auf Basis der Herstellerangaben, ob mit dem Einsatz der gelisteten DiGA zusätzliche ärztliche Leistungen verbunden sind. Ärzte und Psychotherapeuten erhalten hierfür eine zusätzliche Vergütung. Der Erweitere Bewertungsausschuss bei der Kassenärztlichen Bundesvereinigung prüft, ob gesonderte Leistungen in den Einheitlichen Bewertungsmaßstab (EBM) aufzunehmen sind.

Das Bewertungsverfahren beginnt mit dem Antrag des Herstellers (oder eines von ihm benannten Verantwortlichen), der über das entsprechende Antragsportal des BfArM eingereicht wird [11]. Am Ende des Verfahrens steht entweder ein positiver Bescheid des BfArM zur vorläufigen bzw. dauerhaften Aufnahme der DiGA in das BfArM-DiGA-Verzeichnis [12] oder eine begründete Ablehnung der Aufnahme. Im Falle einer Ablehnung kann ein neuer Antrag mit Adressierung der Ablehnungsgründe gestellt werden. Zusätzlich bietet das BfArM immer an, die Punkte, die zur Ablehnung des Antrags geführt haben, in einem Beratungsgespräch zu erläutern und mögliche Lösungsansätze für die Neueinreichung zu empfehlen.

Nach erfolgreicher Aufnahme in das DiGA-Verzeichnis schließen sich die Preisverhandlungen zwischen Hersteller und GKV-Spitzenverband an. Das BfArM ist in Prozessen zu Preisgestaltung und -verhandlung nicht eingebunden.

### Pflichten nach der Listung im Verzeichnis

Wesentliche Änderungen, die einen Einfluss auf die Funktionsweise der DiGA, auf die Bewertungsentscheidung des BfArM oder die Angaben im Verzeichnis haben, sind dem BfArM anzuzeigen, das dann prüft, ob mit deren Umsetzung nach wie vor die Voraussetzungen zur Listung im Verzeichnis gegeben sind oder die DiGA gestrichen werden muss. Von dieser Anzeige und Entscheidung zur weiteren Listung unabhängig sind die medizinprodukterechtlichen Verpflichtungen für den Hersteller, alle Risikoinformationen zu seinem Produkt unverzüglich zu bewerten, u. a. schwerwiegende Vorkommnisse an das BfArM zu melden und ggf. Korrekturmaßnahmen an der DiGA vorzunehmen.

### Anforderungen an eine DiGA: Von Datenschutz über Benutzerfreundlichkeit zu positiven Versorgungseffekten

Wesentlicher Bestandteil des Antrags ist die Bestätigung des Herstellers, dass die DiGA die in den §§ 3 bis 6 sowie den Anlagen der DiGAV definierten Anforderungen an Sicherheit, Funktionalität, Datenschutz und Informationssicherheit sowie Qualität bzw. Benutzerfreundlichkeit, darunter insbesondere Interoperabilität und Barrierefreiheit, erfüllt. Diese Aspekte sind mehrheitlich bereits unabhängig vom DiGA-Fast-Track z. B. für die Konformitätsbewertung bzw. CE-Kennzeichnung des Medizinproduktes oder durch datenschutzrechtliche Vorgaben zu erfüllen.

**Figure 3:**
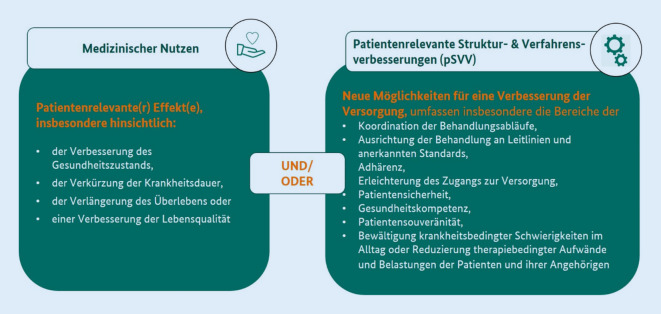

Der zweite Schwerpunkt der Bewertung liegt auf dem Nachweis eines mit der DiGA verbundenen positiven Versorgungseffektes (pVE): Dieser neu mit dem DVG eingeführte Begriff umfasst zum einen „medizinischen Nutzen“, zum anderen „patientenrelevante Struktur- und Verfahrensverbesserungen“ (pSVV), d. h. Parameter, die sich unmittelbar auf die Patienten beziehen (Abb. 3). Dies bedeutet, dass eine App oder z. B. auch Webanwendung dazu beitragen kann, die Gesundheitskompetenz zu fördern, die Koordinierung von Behandlungsabläufen zu verbessern oder Therapieaufwände zu reduzieren.

Für die Angabe des positiven Versorgungseffektes ist die Patientengruppe, d. h. die Indikation in Form von ICD-10-Codes, anzugeben und der entsprechende Nachweis für diese Patientengruppe zu führen. Dieser ist gemäß DiGAV durch quantitative vergleichende Studien zu erbringen. Dabei kommen je nach Fragestellung bzw. postuliertem Effekt sowohl klinische als auch epidemiologische Studien, Methoden der Versorgungsforschung, der Sozial- oder der Verhaltensforschung in Betracht. Dem Zweck der DiGA entsprechend, alltagsnahe und individuelle Therapieunterstützung zu leisten, sind Studien zu DiGA möglichst in der Versorgungsrealität angesiedelt und mithilfe der Erhebung und Aufbereitung versorgungsnaher Daten durchzuführen. Der Vergleich ist gem. DiGAV gegen eine Nichtanwendung der DiGA unter Berücksichtigung der spezifischen Versorgungsrealität (ggf. Wartezeit, Therapiealternativen) zu führen; dies kann intraindividuell im Vorher-nachher-Vergleich oder gegen einen etablierten Therapiestandard erfolgen. Es ist möglich, auf retrospektive Datenauswertungen, z. B. aus Patientenakten, Abrechnungsdaten, Registern oder der Anwendung der DiGA im Versorgungsalltag, zurückzugreifen oder prospektiv ausgerichtete Vergleichsstudien vorzulegen. Ausschlaggebend ist, dass die gewählte Methodik für die zugrunde liegende Fragestellung (Endpunkte, Effekt etc.) geeignet ist.

## Das DiGA-Verzeichnis – Ein zielgruppenspezifisch strukturiertes Webportal mit hoher Transparenz

Neben der systematischen Bewertung der DiGA wird mit dem Fast-Track ein weiteres zentrales Ziel verfolgt: umfassende Transparenz zu den auf dem Markt befindlichen und durch das BfArM nach § 139e SGB V positiv bewerteten digitalen Angeboten. Damit wird dem Wunsch und Bedarf Rechnung getragen, sich umfassend über verfügbare Angebote, insbesondere zu deren Eigenschaften und Leistungsfähigkeit, informieren und diese auch untereinander vergleichen zu können. Zudem können auch Leistungserbringer und Kostenträger auf für sie zugeschnittene Informationen zugreifen, die z. B. für die Verschreibung bzw. für Kostenerstattungsaspekte der DiGA relevant sind.

Das BfArM stellt dafür auf seinen Webseiten das Verzeichnis zu den DiGA nach § 139e SGB V tagesaktuell als nutzerfreundliches und zielgruppenspezifisch strukturiertes Webportal zur Verfügung [12]. Um zu gewährleisten, dass die für die jeweilige Adressatengruppe relevanten Informationen verständlich und zielführend aufbereitet sind, steht das BfArM in intensivem Austausch mit den jeweiligen Interessenvertretern und hat eine Feedbackmöglichkeit implementiert, die zur kontinuierlichen Verbesserung des Verzeichnisses beiträgt.

### Inhalte des Verzeichnisses

Das DiGA-Verzeichnis bietet einen transparenten Überblick zu allen vom BfArM positiv bewerteten DiGA und stellt dazu jeweils Informationen bereit, die in separaten Ansichten für die jeweilige Adressatengruppe verfügbar sind, z. B.:

- grundlegende Informationen,
- Angaben zur Wirkweise sowie den positiven Versorgungseffekten einschließlich der zum Nachweis vorgelegten Evidenz und deren Bewertung durch das BfArM,
- technische Eigenschaften,
- mit der Anwendung der DiGA verbundene Kosten sowie
- Verschreibungsmöglichkeiten und ggf. zusätzlich erforderliche ärztliche Leistungen.

Nutzerfreundliche *Such- und Filterfunktionen* gewährleisten das einfache Finden spezifischer Informationen im Verzeichnis und unterstützen Anwender und Ärzte. Sie umfassen z. B. Suchoptionen und Einstellungen, die eine Filterung nach Indikation, Altersgruppen oder z. B. nach der erforderlichen technischen Plattform ermöglichen. Diese Such- und Filterfunktionen werden sukzessive ergänzt bzw. angepasst.

Im DiGA-Verzeichnis können zunächst *grundlegende Informationen* (Basisdaten) wie der Name der DiGA sowie Angaben zum Hersteller, zur medizinischen Zweckbestimmung nach Medizinprodukterecht und zur Gebrauchsanweisung für die DiGA nach Medizinprodukterecht eingesehen werden.

*Technische Informationen* helfen DiGA-Nutzern, vor einer Verordnung der DiGA zu prüfen, ob diese mit ihrem Mobilgerät oder Computer genutzt werden kann. Ferner unterstützen diese Angaben bei der Einschätzung, ob die Daten der DiGA in einem geeigneten Format ausgegeben werden können, um bei Bedarf mit anderen Geräten oder Anwendungen ausgetauscht werden zu können (Interoperabilität).

Das Verzeichnis bietet zudem im Bereich „Informationen für Versicherte und Patienten“ relevante Informationen zu der DiGA und ihrer Anwendung in allgemeinverständlicher Beschreibung aus der Nutzerperspektive, insbesondere:

- zur Zielsetzung, Wirkungsweise, Funktionen und Nutzung der DiGA,
- zum positiven Versorgungseffekt,
- zum Datenschutz und zur Informationssicherheit,
- zu eventuellen Mehrkosten, z. B. für Zubehör, sowie
- zu Voraussetzungen für die Nutzung der DiGA, beispielsweise Hardwareanforderungen.

Die im Verzeichnis unter „Informationen für Fachkreise“ enthaltenen Angaben für Leistungserbringer (z. B. Ärzte, Psychotherapeuten) sollen diese dabei unterstützen, gemeinsam mit den Patienten die jeweils am besten geeignete DiGA auszusuchen und zu verordnen. Auch sollen sie erkennen können, ob mit der Verordnung der DiGA weitere ärztliche Leistungen verbunden sind. Dafür werden z. B. folgende Angaben bereitgestellt:

- Information zur dauerhaften oder vorläufigen Aufnahme in das Verzeichnis,
- Patientengruppe/Indikation, für die positive Versorgungseffekte nachgewiesen wurden,
- empfohlene Mindest- und eventuell Höchstdauer der Nutzung,
- Kosten der Übernahme durch die GKV,
- Angaben zu den vorgelegten wissenschaftlichen Nachweisen sowie
- Quellen für die in der DiGA umgesetzten medizinischen Inhalte und Verfahren.

### Interoperabilität: Maschinenlesbare Schnittstelle zum DiGA-Verzeichnis

Auch für das Verzeichnis spielt Interoperabilität eine zentrale Rolle. So ermöglicht eine Programmierschnittstelle (Application Programming Interface, API) den tagesaktuellen Abruf aller Angaben aus dem Verzeichnis in Praxisverwaltungssysteme für Verordnungs- und Abrechnungsprozesse [13]. Weitere Antragsberechtigte, wie z. B. medizinische Fachgesellschaften, Krankenkassen, Ärzteverbände, Forschungsinstitutionen oder Patientenverbände, erhalten durch den direkten Zugriff auf die Daten die Möglichkeit, ergänzende Empfehlungen an ihre jeweiligen Zielgruppen zu geben und damit informierte Nutzungsentscheidungen in der Breite zu unterstützen.

## Unterstützung durch das BfArM

Um Herstellern mit Blick auf dieses neue Bewertungsverfahren und damit verbundene Anforderungen im Vorfeld einer Antragsstellung Planungssicherheit zu geben, stellt das BfArM ein umfassendes Informations- und Unterstützungsangebot zur Verfügung: Angefangen mit einem Leitfaden [14] über ergänzende FAQ sowie aktuelle Informationen zum Verfahren auf der Website des BfArM [15], einer Ausfüllhilfe für das Antragsportal reicht das Angebot bis hin zu diversen (teilweise gebührenpflichtigen) Beratungsformaten mit unterschiedlichem Detailierungsgrad. Zudem bietet das BfArM auch ein umfassendes Informationsangebot, das Orientierung zu wesentlichen Grundlagen, wie zum Beispiel zu medizinprodukterechtlichen Themen oder zur Einordnung von Produkten als Medizinprodukt [3], gibt.

### DiGA-Leitfaden

Das BfArM hat die gesetzlichen Grundlagen und Anforderungen, die an verschiedenen Stellen im SGB V und in der DiGAV, inkl. Anlagen, dargelegt sind, in einem umfassenden Leitfaden zusammengefasst [14]. In diesem werden die Antragsverfahren und erforderlichen Nachweise zur vorläufigen oder dauerhaften Aufnahme sowie zur Anzeige einer wesentlichen Änderung in allen Schritten erläutert. So legt das BfArM u. a. anhand zahlreicher Beispiele ausführlich dar, wie es die normativen Vorgaben zur Aufnahme in seiner Bewertungspraxis auslegt. Damit wird größtmögliche Transparenz bezüglich der konkret zu erfüllenden Anforderungen geschaffen. Der Leitfaden ist als lebendes Dokument konzipiert, das kontinuierlich aktualisiert und weiterentwickelt wird.

### Beratung über das BfArM-Innovationsbüro

Das Innovationsbüro [6] ist eine erste Anlaufstelle für Hersteller und bietet ein breites Spektrum an Unterstützung. So beantwortet das Innovationsbüro z. B. einfache Verfahrensfragen unkompliziert und unbürokratisch durch telefonische oder schriftliche Auskunft.

Im Rahmen von Kick-off-Meetings können Projektideen in frühen Entwicklungsstadien ergebnisoffen und informell mit dem BfArM diskutiert werden. Ziel ist es, Antragsstellende frühzeitig über regulatorische Rahmenbedingungen und Voraussetzungen aufzuklären und eine orientierende Hilfestellung zum DiGA-Fast-Track zu geben.

Komplexere Fragen zur DiGA-Konformität, Interoperabilität, Datenschutz bis hin zu Fragestellungen zur produktbezogenen Nachweisführung des positiven Versorgungseffekts werden in interdisziplinären Teams mit Experten des BfArM in einer „DiGA-Beratung“ diskutiert.

## Erfahrungen und erste Bilanz

**Figure 4:**
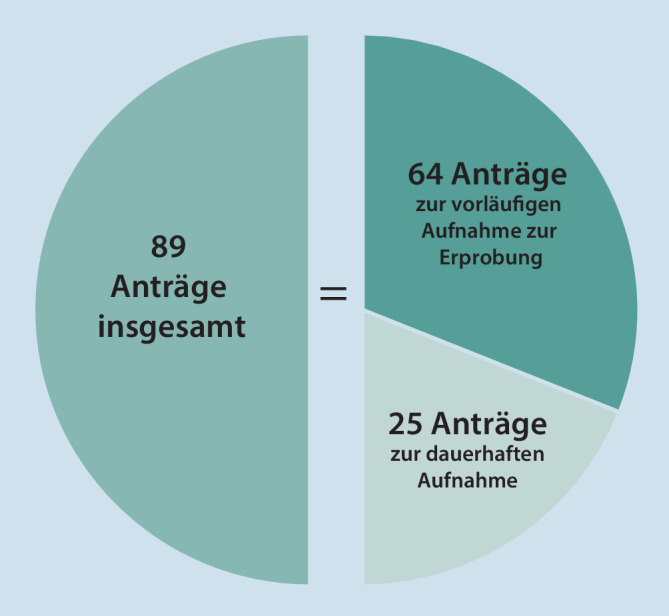

Insgesamt ist festzustellen, dass der DiGA-Fast-Track, das diesbezügliche Beratungsangebot und die vom BfArM bereitgestellten unterstützenden Informationen auf großes Interesse gestoßen sind und rege in Anspruch genommen werden. Eine quantitative Übersicht zu den bislang eingegangenen Anträgen ist in Abb. 4 dargestellt.

**Figure 5:**
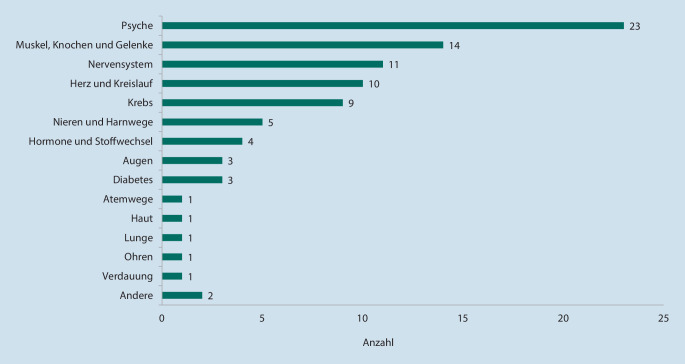

Dabei sind die eingereichten Anträge bislang vor allem im Bereich psychischer Erkrankungen angesiedelt, weitere DiGA beziehen sich u. a. auf Erkrankungen des Nerven- oder Skelettsystems (Abb. 5).

Mit Stand 18.08.2021 wurden insgesamt 20 DiGA im Verzeichnis veröffentlicht, 4 Anträge negativ beschieden und 42 Anträge vom Antragsteller während des Bewertungsverfahrens zurückgezogen. Im Fall der Ablehnung oder des Rückzugs eines Antrages waren unterschiedliche Gründe ausschlaggebend. In einigen Verfahren betraf dies Mängel hinsichtlich des Datenschutzes und/oder der Informationssicherheit. In den meisten Fällen konnten die Hersteller jedoch keine ausreichenden Nachweise für die postulierten positiven Versorgungseffekte vorlegen.

Bei den zurückgezogenen Anträgen zur dauerhaften Aufnahme ist festzustellen, dass die mit dem Antrag vorgelegten Daten z. T. aus zurückliegenden Studien zu anderen Zwecken stammten, die oftmals nicht nach den Kriterien der evidenzbasierten Medizin durchgeführt wurden. Die Studien wiesen teilweise gravierendere Limitationen auf; so existierten z. B. nur unvollständige oder keine der gem. DiGAV geforderten Studiendokumente (z. B. Studienberichte, Studienprotokolle). Neben diesen grundlegenden methodischen Mängeln konnten die Ergebnisse solcher Studien darüber hinaus oft nicht zeigen, dass die Anwendung der DiGA Vorteile gegenüber ihrer Nichtanwendung brachte.

Bei den zurückgezogenen Anträgen auf vorläufige Aufnahme sind die Ursachen überwiegend auf unzureichende systematische Datenauswertungen zurückzuführen, die z. B. einen für den mit der Anwendung angestrebten positiven Versorgungseffekt deutlich zu kurzen Beobachtungszeitraum hatten und/oder eine zu geringe Probandenzahl vorwiesen oder nicht die postulierten positiven Versorgungseffekte aufzeigten.

**Figure 6:**
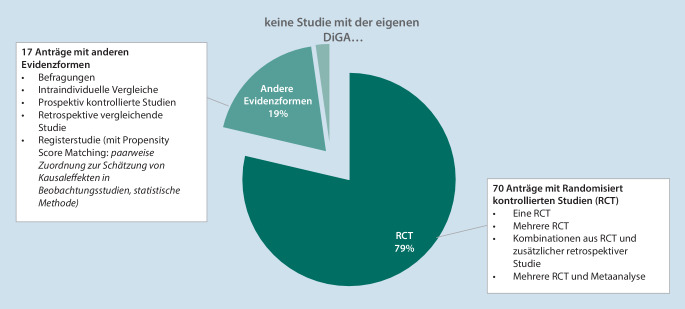

Der Schwerpunkt der eingereichten Evidenznachweise liegt klar bei randomisierten kontrollierten Studien, wobei im Hinblick auf den besonderen Charakter der DiGA durchaus auch andere Evidenzformen eingereicht wurden (Abb. 6).

Darüber hinaus waren im Rahmen der Antragstellung weitere Herausforderungen zu erkennen, wie z. B. die Erfüllung der Anforderungen an Interoperabilität, Datenschutz und Informationssicherheit. Insbesondere die Auswirkungen des Urteils des Europäischen Gerichtshofs vom 16.07.2020 („Schrems II-Urteil“) führten dazu, dass Hersteller teilweise kurzfristig und innerhalb des laufenden Bewertungsverfahrens ihre datenverarbeitenden Dienstleister wechseln mussten. Mit der Weiterentwicklung auch des gesetzlichen Rahmens zu diesem Thema, z. B. durch die Einführung entsprechender Zertifikate mit dem DVPMG, erwarten wir zusätzliche Klarheit für Hersteller und damit auch verbesserte Angebote durch datenverarbeitende Dienstleister.

Es zeigte sich außerdem, dass das auf max. 3 Monate begrenzte Bewertungsverfahren durchaus auch eine Herausforderung für Hersteller darstellen kann. In dem vom Gesetzgeber bewusst als Fast-Track angelegten Verfahren werden Mängel und offene Fragen seitens des BfArM so früh wie möglich an den Antragsteller kommuniziert, um ihm Gelegenheit zur Stellungnahme und Behebung der Mängel zu geben. Aufgrund des fehlenden Clock Stop ist dies allerdings mit engen Fristen verbunden, um auch die nachfolgende Bewertung der Stellungnahme zu ermöglichen. Es ist daher wichtig, dass Antragsteller während des Bewertungsprozesses kurzfristig ansprechbar und in der Lage sind, schnell fachlich fundierte Stellungnahmen z. B. zur statistischen Planung abzugeben.

Insgesamt erlauben die bisherigen Erfahrungen aus den Beratungen und Bewertungen eine positive Bilanz. Der enge Austausch zwischen BfArM und Antragstellern ermöglicht es in vielen Fällen, Mängel an den Anträgen kurzfristig bzw. bereits im Vorfeld auszuräumen. Gerade im Hinblick auf die Evidenz zum Nachweis des positiven Versorgungseffekts bewertet das BfArM im Sinne der Ziele und Vorgaben der DiGAV mit Augenmaß, stellt aber zugleich sicher, dass die in das DiGA-Verzeichnis aufgenommenen und damit durch die GKV erstattungsfähigen Anwendungen alle erforderlichen Nachweise erbringen und diese den einschlägigen wissenschaftlichen Standards entsprechen.

## Fazit und Ausblick

Mit dem DiGA-Fast-Track beim BfArM und der Einführung patientenrelevanter Struktur- und Verfahrensverbesserungen als neuer Bewertungsdimension neben dem medizinischen Nutzen hat der Gesetzgeber einen wichtigen, zukunftsorientierten Schritt zur Etablierung qualitätsgesicherter digitaler Gesundheitsanwendungen in der Gesundheitsversorgung in Deutschland getan. Das Interesse zeigt, dass Deutschland eine Vorreiterrolle in diesem innovativen Bereich eingenommen hat und auch außerhalb Deutschlands die digitale Entwicklung genau verfolgt wird.

Das DiGA-Verzeichnis und die dortige ausführliche Darlegung der wissenschaftlich fundierten Bewertungsentscheidungen des BfArM schaffen Übersicht, Transparenz und Vergleichbarkeit sowohl hinsichtlich der Sicherheits- und Leistungsfähigkeit und des Datenschutzes als auch bzgl. des positiven Versorgungseffektes und der dafür vorgelegten Evidenz. Leistungserbringer, Patienten und andere Interessierte können sich damit ein umfassendes Bild zu den bewerteten DiGA und dem entsprechenden Mehrwert machen.

Die noch deutliche Zahl an Rücknahmen im Bewertungsprozess zeigt, dass in vielen Fällen die zeitlichen und inhaltlichen Herausforderungen des Fast-Track-Verfahrens bzw. die Anforderungen aus Gesetz und Verordnung von den Antragstellern unterschätzt bzw. die umfangreichen Beratungsangebote des BfArM noch nicht ausreichend genutzt wurden. Dies betrifft insbesondere Fragen des Datenschutzes, der Datensicherheit und des wissenschaftlichen Nachweises zum positiven Versorgungseffekt. Gemessen an den Anfragen und Beratungen ist die Zahl der im DiGA-Verzeichnis gelisteten DiGA noch verhältnismäßig gering. Einen weiteren Einflussfaktor stellen hier die strategischen Überlegungen der Hersteller dar; diese äußern mitunter in Gesprächen, die ersten Bewertungen (zu den eigenen DiGA) sowie insbesondere die ersten Ergebnisse der Preisverhandlungen und damit auch letztendlich verknüpfte Markt(durchdringungs)chancen abzuwarten, ehe sie weitere Produkte über den DiGA-Fast-Track in die Regelversorgung einbringen oder sich für Alternativen in Form von z. B. Selektivverträgen mit einzelnen Krankenkassen entscheiden.

Hier möchte das BfArM zukünftig noch besser im Vorfeld der Antragstellung unterstützen und seine Erfahrungen aus den bisherigen Verfahren noch stärker in Form konkreter Hinweise vermitteln. Die bereits angebotenen Informationen zum Antragsverfahren werden kontinuierlich fortgeschrieben und durch weitere Dokumente, wie z. B. Checklisten zur Studienplanung oder Erläuterungen zum Evaluationskonzept, ergänzt.

Bei der Einführung des DiGA-Fast-Track hat der Gesetzgeber bewusst die Antragstellung auf Medizinprodukte niedriger Risikoklassen (I und IIa) begrenzt, um damit zunächst Erfahrungen zu sammeln. Eine logische Weiterentwicklung mit Blick auf die positiven Ergebnisse und die zu erwartende Höherklassifizierung der meisten DiGA in den kommenden Jahren sollte daher auch die Einbeziehung höherer Risikoklassen oder beispielsweise auch von digitalen In-vitro-Diagnostika nach der Verordnung (EU) 2017/746 (IVDR) sein.

Mit dem Digitale Versorgung und Pflege – Modernisierungs-Gesetz (DVPMG) [16] nimmt der Gesetzgeber als erste Ausbaustufe die Unterstützung der Pflege durch digitale Pflegeanwendungen (DiPA) in den Fokus und etabliert ein entsprechendes Bewertungsverfahren zur Kostenerstattung. Auch die wichtigen Themen Datenschutz bzw. Informationssicherheit und Interoperabilität erfahren durch die Entwicklung von Zertifikaten und ein zu etablierendes Schnittstellenverzeichnis erste Weiterentwicklungen und Optimierungen des Verfahrens. Das BfArM begrüßt diesen nächsten innovativen Schritt und bereitet sich gemeinsam mit dem BMG intensiv auf diese wichtigen weiteren Aufgaben vor, um sich auch hier proaktiv für die Unterstützung der digitalen Gesundheitsversorgung in Deutschland einzusetzen.
